# *TaNBP1*, a guanine nucleotide-binding subunit gene of wheat, is essential in the regulation of N starvation adaptation via modulating N acquisition and ROS homeostasis

**DOI:** 10.1186/s12870-018-1374-6

**Published:** 2018-08-13

**Authors:** Zhipeng Liu, Yingjia Zhao, Xiaoying Wang, Mengya Yang, Chengjin Guo, Kai Xiao

**Affiliations:** College of Agronomy, Key Laboratory of Crop Growth Regulation of Hebei Province, Agricultural University of Hebei, Hebei, 071001 China

**Keywords:** Wheat (*Triticum aestivum* L.), Guanine nucleotide-binding protein subunit beta, G-protein, Gene expression, N starvation stress, Functional characterization

## Abstract

**Background:**

Nitrate (NO_3_^−^) is the major source of nitrogen (N) for higher plants aside from its function in transducing the N signaling. Improving N use efficiency of crops has been an effective strategy for promotion of the sustainable agriculture worldwide. The regulatory pathways associating with N uptake and the corresponding biochemical processes impact largely on plant N starvation tolerance. Thus, exploration of the molecular mechanism underlying nitrogen use efficiency (NUE) and the gene wealth will pave a way for molecular breeding of N starvation-tolerant crop cultivars.

**Results:**

In the current study, we characterized the function of *TaNBP1*, a guanine nucleotide-binding protein subunit beta gene of wheat (*T. aestivum*), in mediating the plant N starvation response. TaNBP1 protein harbors a conserved W40 domain and the TaNBP1-GFP (green fluorescence protein) signals concentrate at positions of cytoplasm membrane and cytosol. *TaNBP1* transcripts are induced in roots and leaves upon N starvation stress and that this upregulated expression is recovered by N recovery treatment. *TaNBP1* overexpression confers improved phenotype, enlarged root system architecture (RSA), and increased biomass for plants upon N deprivation relative to the wild type, associating with its role in enhancing N accumulation and improving reactive oxygen species (ROS) homeostasis. Nitrate transporter (NRT) gene *NtNRT2.2* and antioxidant enzyme genes *NtSOD1*, *NtSOD2*, and *NtCAT1* are transcriptionally regulated under *TaNBP1* and contribute to the improved N acquisition and the increased AE activities of plants.

**Conclusions:**

Altogether, *TaNBP1* is transcriptional response to N starvation stress. Overexpression of this gene enhances plant N starvation adaptation via improvement of N uptake and cellular ROS homeostasis by modifying transcription of NRT gene *NtNRT2.2* and antioxidant enzyme genes *NtSOD1*, *NtSOD2*, and *NtCAT1*, respectively. Our research helps to understand the mechanism underlying plant N starvation response and benefits to genetically engineer crop cultivars with improved NUE under the N-saving cultivation conditions.

**Electronic supplementary material:**

The online version of this article (10.1186/s12870-018-1374-6) contains supplementary material, which is available to authorized users.

## Background

Nitrogen (N) is an essential macronutrient for the plant growth and development. For a long time, increased input of the N fertilizers has contributed greatly to the improvement of the crop productivity. However, overdose of N input during crop production has also caused serious environment problem in addition to the increased investment [1]. Improvement of N use efficiency (NUE) of crops under the N-saving cultivation conditions has thus been an effective strategy for sustainable agriculture given that it can alleviate the N-associated environmental pollution.

Plants have evolved multifaceted strategies in response to external N availabilities [2, 3]. Upon N deficiency, plants respond to the N-starvation signaling via initiation of response pathways involving transcriptional modulation of a suite of N-starvation responsive genes, whose synergic action contributes to the plant N starvation adaptation. Of which, distinct transcription factors (TFs) and nitrate transporters (NRTs) have been confirmed to be involved in the N starvation response. For example, the TF encoding genes, including Arabidopsis nitrate regulated 1 gene (*ANR1*) and nodule inception-like protein 7 gene (*NLP7*) as well as maize DNA finger binding protein gene (*ZmDOF1*), act as crucial mediators in the regulation of N starvation tolerance via mediating external nitrate response and internal N assimilation [4–6]. The Arabidopsis NRT genes (i.e., *CHL1* and *NRT2.1*) play important roles in sensing nitrate signal, taking up external nitrate, and tanslocating internal N across tissues [7, 8]. It is now well documented that plants are able to sense external nitrate availability. Therefore, nitrate acts as a signal molecule in plants involving regulation of many biological processes associated with N intake, metabolism, and related gene expression [9]. However, although a large set of investigations performed focuses on understanding of the N starvation responses and adaptation pathways, detailed mechanisms as to how plants perceive and transduce the N signaling still remain largely unknown.

GTP-binding proteins (G-proteins) are heterotrimeric consisting of three subunits, Gα, Gβ, and Gγ. In plant species as well as other eukaryotes, proteins in this functional class play essential roles in transducing the signals initiated by internal cues as well as environmental stressors [9, 10]. The subunits constituting G-protein execute distinct functions across diverse biological processes. For example, Arabidopsis dimer Gβγ1 and Gβγ2 involves plant defensiveness to a variety of fungal pathogen infection [11]; it acts as an essential mediator in preventing plant infection and injury from *P. syringae*, a kind of bacterial pathogen, via regulating programmed cell death (PCD) and reactive oxygen species (ROS) homeostasis [10]. Likewise, subunit Gα of the G-protein endows plants enhanced defensiveness against *P. syringae* infection and injury through adjustment of stomata movement [12]. Several Gα-like subunits have been validated to be involved in establishing extra-large G proteins referred to as XLGs with subunits Gβ and Gγ, conferring plants increased GTP hydrolysis capacity by which to promote the substrate degradation and modulate the flowering characterization of plants [13, 14].

Subunit Gβ involves the constitution of the G-protein and exerts similar biological functions to Gα and Gγ, two other G-protein subunits. It is also involved in plant pathogen defensiveness [15]. In addition, this subunit has also been an essential mediator in multiple physiological processes, including root gravitropic response [16], tissue differentiation [17], and channel-mediated ion transportation [18]. These findings suggest the diverse roles of subunit Gβ in regulating plant growth, development, and abiotic stress responses.

Wheat (*T. aestivum* L.) is one of the important cereal crops cultivated around the world. Thus far, a large set of investigations has been performed focusing on understanding of the physiological and biochemical mechanisms underlying plant N starvation response [19]. However, the molecular networks associating with N starvation signaling perceiving and transducing still remain largely unknown in the *T. aestivum* species. Previously, based on microarray analyses, we identified a guanine nucleotide-binding protein (NBP) encoding *TaNBP1* gene (GenBank accession No. AK332651), a beta subunit (Gβ) gene for G-protein of wheat, to be upregulated in expression under N starvation condition. In this study, we functionally characterized *TaNBP1*, a subunit Gβ encoding gene of wheat, in regulating the N starvation stress tolerance. Our results indicate that *TaNBP1* is N-starvation inducible and acts as an essential modulator in plant N deficiency tolerance through regulating N acquisition and cellular ROS homeostasis.

## Methods

### Characterization analysis of *TaNBP1*

Sequence similarities of TaNBP1 and its homologous proteins in plant species were determined using MEGA7 software (https://www.mega.com). Conserved domain shared by the NBP proteins including TaNBP1 and its plant counterparts was specified as previously described [20]. Phylogenetic relationship among *TaNBP1* and its homologous genes was established using the DNAStar software (https://www.dnastar.com).

### 3-D structure prediction and subcellular localization analysis on TaNBP1

An online tool referred to as SWISS-MODEL algorithm (https://swissmodel.expasy.org/interactive) that simulates protein three-dimensional structure was adopted to predict the 3-D structure of TaNBP1. To define the subcellular localization of TaNBP1 sorted from endoplasmic reticulum (ER), we generated an expression cassette harboring a *TaNBP1*-*GFP* (green florescence protein encoding gene) fusion gene as previously described [21], in which, the coding sequence (CDS) of *TaNBP1* was amplified by RT-PCR using specific primer pairs (Additional file 1: Table S1) and integrated in frame with *GFP* under control of the constitutive CaMV35S promoter. GFP signals derived from the fusion in transformed tobacco epidermis cells were detected as described by Guo et al. (2013) [21].

### Expression analysis of *TaNBP1*

Shiyou 20, an elite high yielding wheat cultivar in North China used in our microarray analyses to identify genes differentially expressed upon N starvation stress, was selected to investigate the expression patterns of *TaNBP1* at two N supplies. With this aim, the seeds were germinated under 25 °C in darkness. After germination, the young seedlings were cultured hydroponically in a growth chamber. To this end, roots of the young seedlings were immersed into standard Murashige and Skoog (MS) solution (16 mM N) through holes of plastic foam that floated on the nutrient solution, which was renewed twice one week and air-circulated by a mini pump. Growth conditions for the seedlings were as follows: photoperiod of 16 h/8 h (light/ dark), temperature of 22 °C, light intensity of 230 μmol m^− 2^ s^− 1^ during light phase, and air humidity of 65 to 75%. At the third leaf-expansion stage, wheat seedling were subjected to N starvation treatment by transferring into a modified MS solution that contained reduced N (0.02 mM N), which was established by reducing NH_4_NO_3_ and KNO_3_ amounts and supplementing KCl in the meantime to sustain unaltered K content in the solution. Additionally, a followed N recovery treatment was initiated by retransferring the 27 h N-deprived seedlings again to a standard MS solution. At 0 h (before N starvation), 1, 3, 9, and 27 h after N starvation and N recovery treatments, root and leaf tissues were collected. Transcripts of *TaNBP1* in both tissues examined were determined based on qRT-PCR. Briefly, total RNA in the tissues was extracted by TRIzol reagents (Invitrogen, USA). After treatment with RNase-free DNase (TaKaRA, Dalian, China) to avoid genomic DNA contamination, the total RNA (2 μg) was subjected to first-strand cDNA synthesis using RT-AMV transcriptase (TaKaRa, Dalian, China) in 20 μL reaction volume using oligo (dT)18 at 42 °C for 30 min, according to the manufacturer’s instructions. qRT-PCR analysis was performed in a total volume of 25 μL containing 12.5 μL of SYBR Premix ExTaqTM (TaKaRa, Dalian, China), 0.5 μL of forward and reverse primers, 1 μL cDNA and 10.5 μL nuclease-free water. *TaNBP1* transcripts in tissues examined were calculated based on the 2^-ΔΔCT^ method using wheat *Tatubulin* as an internal control. The gene-specific primers used for qRT-PCR analysis are shown in Additional file 1: Table S1.

### Generation of transgenic *N. benthamian* lines with *TaNBP1* overexpression

Transgenic *N. benthamian* lines with *TaNBP1* overexpression were generated to characterize the gene function in mediating N starvation tolerance. With this aim, an expression cassette harboring the *TaNBP1* CDS was constructed using conventional approach. Briefly, the *TaNBP1* CDS was amplified by RT-PCR with specific primers (Additional file 1: Table S1), then inserted into the *Nco*I/*BstE*II restriction sites in binary vector pCAMBIA3301 at position downstream the CaMV35S promoter. Genetic transformation of the expression cassette into *A. tumefaciens* stain EHA105 and further generation of the *N. benthamian* lines with *TaNBP1* overexpression were performed as described previously [22].

### Assays of phenotypes and biomass of transgenic lines under different N treatments

Two lines with higher *TaNBP1* expression levels (Lines 2 and 3, Additional file 1: Figure S1A) were selected to address the gene function in mediating the plant N starvation response. To this end, seeds of Lines 2 and 3 at T3 generation were germinated in darkness. Ten days after germination, uniform seedlings of the transgenic and wild type (WT) were hydroponically cultured in standard MS solution for normal growth (16 mM N) or subjected to N starvation treatment by growing under modified MS solution containing reduced N (0.06 mM N); this modified deficient N solution was prepared similarly to that for N-deprived wheat seedlings. The transgenic and WT seedlings under contrasting N conditions were cultured under same growth conditions as wheat seedlings. During culture process, nutrient solutions were air-circulated using a mini pump and renewed twice within each week. Six weeks later, phenotypes of the transgenic and WT plants were recorded using a digital camera and the biomass were obtained after drying the plant samples in an oven at 80 °C for 48 h.

### Assays of N concentrations and NRT gene expression patterns in N-deprived transgenic lines

N concentrations and the nitrate transporter (NRT) gene expression patterns in the transgenic lines (Lines 2 and 3) and WT were evaluated after the N starvation treatment. Of which, the N concentrations were assessed as described previously (Guo et al. 2011) [23]. Plant N accumulative amounts in transgenic lines and WT were calculated by multiplying N concentration and plant biomass. To characterize the NRT genes that putatively involve the mediation of N uptake and internal N translocation across tissues, a set of NRT encoding genes of tobacco, including *NtNRT1.1-s*, *NtNRT1.1-t*, *NtNRT1.2-s*, *NtNRT1.1–2.t*, *NtNRT2;1*, and *NtNRT2.2*, were subjected to expression evaluation using the N-deprived *TaNBP1* overexpression lines and WT as samples, based on semiquantitative RT-PCR or qRT-PCR [21]. Tobacco constitutive gene referred to as *Nttubulin* was used for normalization of the NRT gene transcripts using specific primers (Additional file 1: Table S1).

### Assays of growth and N-associated traits in *NtNRT2;2* overexpression lines

*NtNRT2.2* was shown to be significantly upregulated in expression in the N-deprived *TaNBP1* overexprresion lines (Lines 2 and 3) compared with wild type, suggesting its involvement in the *TaNBP1*-mediated N starvation response. To evaluate the function of this NRT gene in mediating plant N uptake, transgenic *N. benthamian* lines with *NtNRT2.2* overexpression were generated using similar procedure for establishment of *TaNBP1* overexpression lines. Primers for amplification of *NtNRT2.2* CDS are shown in Additional file 1: Table S1. To define the *NtNRT2.2*-mediated N acquisition characterization, NtNRT2.2–1 and NtNRT2.2–2, two lines with high expression of this NRT gene (Additional file 1: Figure S1B), were subjected to cultivation under N starvation treatment. With this purpose, seeds of NtNRT2.2–1 and NtNRT2.2–2 at T3 generation were germinated in darkness. Ten days after germination, uniform seedlings of the transgenic and wild type (WT) were hydroponically cultured in standard MS solution for normal growth (16 mM N) or subjected to N starvation treatment by growing under modified MS solution containing reduced N (0.06 mM N), which was established to be same for N-deprived WT and lines with *TaNBP1* overexpression as aforementioned. Likewise, nutrient solutions were air-circulated using a mini pump and renewed twice within each week during the culture process. Phenotypes, biomass and N concentrations were assessed six weeks later followed the aforementioned description.

### Assessments of ROS parameters and expression patterns of antioxidant related genes in *TaNBP1* overexpression lines

Given the close relation between cellular reactive oxygen species (ROS) homeostasis and plant N starvation response, we assessed the activities of a set of antioxidant enzymes (AEs), including superoxide dismutase (SOD), catalase (CAT), and peroxidase (POD), and the contents of malondialdehyde (MDA), hydrogen peroxide (H_2_O_2_) and superoxide anion in the N-deprived *TaNBP1* overexpression lines (Lines 2 and 3) and wild type. The parameters described above were determined as reported by Huang et al. 2010 [24]. To define genes possibly involving regulation of the *TaNBP1*-mediated SOD, CAT, and POD activities, a suite of AE genes of tobacco, including five encoding SOD proteins (i.e., *NtSOD1*, *NtSOD2*, *NtSOD3*, *NtMnSOD1*, and *NtMnSOD2*), six coding for CAT proteins (i.e., *NtCAT*, *NtCAT1*, *NtCAT3*, *NtCAT1;1*, *NtCAT1;2*, and *NtCAT1;3*), and eleven encoding POD proteins (i.e., *NtPOD1;1* to *NtPOD1;7*, *NtPOD2;1*, *NtPOD2;2*, *NtPOD4*, and *NtPOD9*), were subjected to expression evaluation using the N-deprived *TaNBP1* overexpression lines based on RT-PCR or qRT-PCR. Accession numbers and primer pairs used for these AE genes are shown in Additional file 1: Table S1.

### Functional analysis of differential AE genes in modulating the AE activities

Expression analysis on the AE encoding genes revealed that three of which, including *NtSOD1*, *NtSOD2*, and *NtCAT1*, were shown to be differentially expressed in the N-deprived *TaNBP1* overexpression lines. To characterize their function in mediating plant AE activities under N starvation conditions, transgenic lines overexpressing these AE genes were generated. With this aim, CDS of the AE genes was PCR amplified and separately inserted into the binary vector pCAMBIA3301 at the *Nco*I/*BstE*II restriction sites under control of the CaMV35S promoter. Constructing expression cassettes harboring these AE genes and genetically transforming them into tobacco were performed to be similar in generating the *TaNRT2;2* overexpression lines as described above. Primer pairs used for amplifying the AE genes are shown in Additional file 1: Table S1.

Two representative lines for each AE gene were selected to define the gene-mediated AE activities under both N normal and N starvation treatments. With this purpose, seeds of WT and transgenic lines at T3 generation with overexpression of the AE genes, including NtSOD1–1 and NtSOD1–2 for *NtSOD1*, NtSOD2–1 and NtSOD2–3 for *NtSOD2*, and for NtCAT1–2 and NtCAT1–3 *NtCAT1*, were germinated in darkness. Ten days after germination, uniform transgenic and WT seedlings were vertically cultured onto agar media containing 1/2 MS salts (8 mM N) or modified 1/2 MS salts with reduced N (0.06 mM N) which was established similarly to that for N-deprived wheat seedlings as aforementioned. In addition, same growth conditions were adopted for culturing these transgenic and WT seedlings. Three weeks after treatments, the transgenic and WT seedlings were subjected to assay of AE activities performed similarly to those for *TaNBP1* overexpression lines.

### Statistical analysis

The mean values of the plant biomass, N concentration, N amount, activities of SOD, CAT, and POD, content of MDA, and qRT-PCR data in WT and transgenic lines under sufficient or deficient N conditions were derived from results of four replicates. Standard errors of the mean values and the significant differences among the mean values were analyzed using the Statistical Analysis System software (SAS Corporation).

## Results

### The characterization of *TaNBP1*

**Fig. 1:**
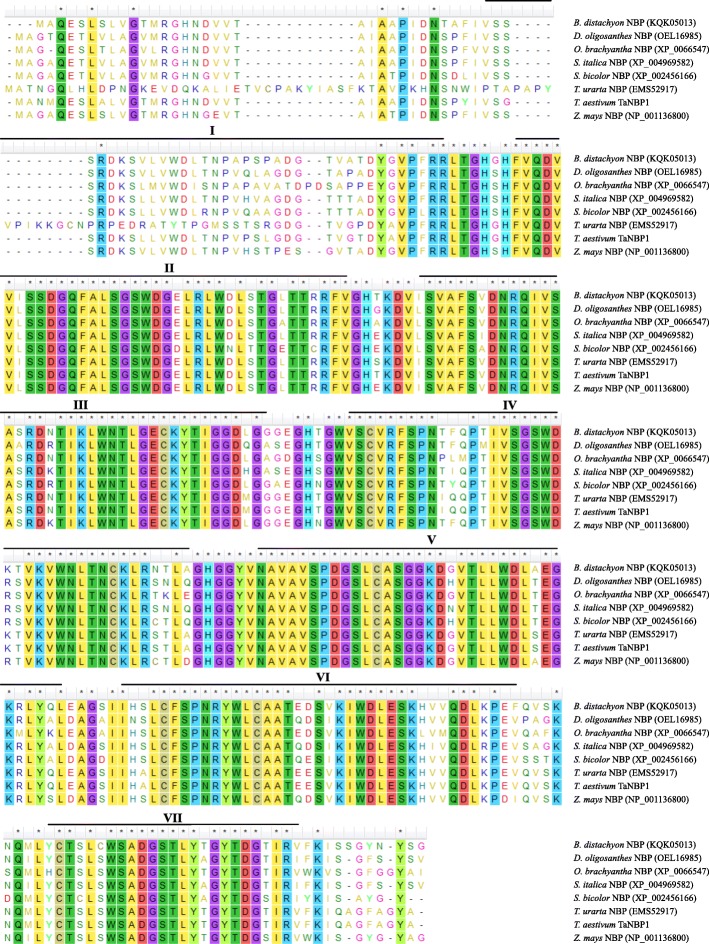
Alignment results between TaNBP1 and the homologous proteins in various plant species Seven conserved WD40 repeat domains are highlighted with lines over amino acid residues and specified by I to VII, in which, I resides at positions of aa 37 to aa 70, II of aa 78 to aa 115, III of aa 121 to aa 160, IV of aa 168 to aa 206, V of aa 213 to aa 247, VI of aa 253 to aa 292, and VII of aa 301 to aa 325

*TaNBP1* cDNA is 1273 bp-long that encodes a 335 aa polypeptide (Additional file 1: Figure S1); the predicted molecular mass and an isoelectric point (pI) of TaNBP1 are 36.28 kD and 6.32, respectively. At amino acid level, TaNBP1 shares high similarities to its counterparts from *B. distachyon*, *D. oligosanthes*, *O. brachyantha*, *S. italica*, *S. bicolor*, *T. urartu*, and *Z. mays*; all of them harbor the conserved WD40 domain involving the constitution of seven blade units (Fig. 1). At nucleic acid level, *TaNBP1* shows high identities to the homologous genes in diverse plant species, with highest similarities to those from *H. vulgare* (AK359815), *B. distachyon* (XM_003567896), *O. sativa* (CT833917), and *S. italica* (XM_004961250) (Additional file 1: Figure S2). These results suggest that *TaNBP1* shared similarly evolved pathway to its plant counterparts.

**Fig. 2:**
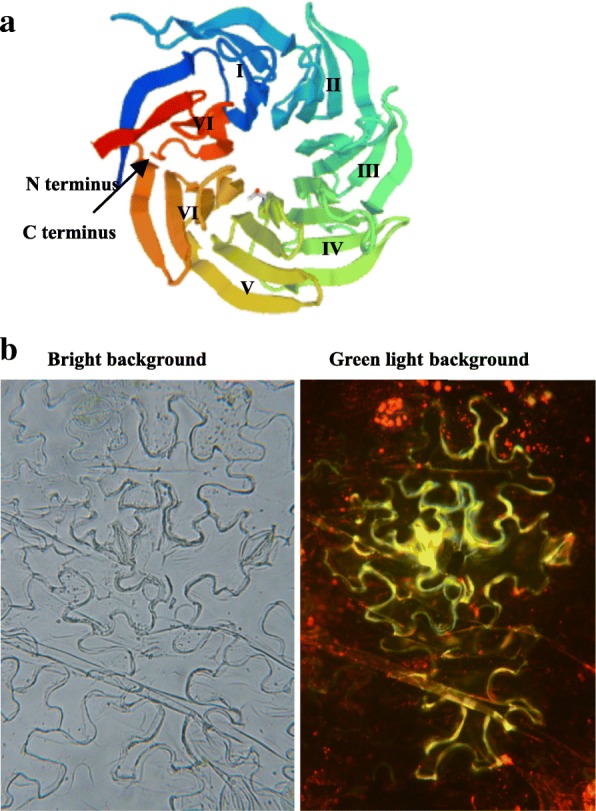
Predicted three dimensional structure of TaNBP1 and its subcellular localization after sorted from endoplasmic reticum. **a** diagram of three dimensional structure of TaNBP1; (**b**) signals derived from TaNBP1-GFP fusion in transformed *N. benthamian* epidermal cells. In (**a**), conserved WD40 repeat domains are specified by I to VII that involve establishing typical sevenfold β-propeller. In (**a**), GFP signals derived from TaNBP1-GFP fusion detected under fluorescence microscopy

Based on three dimensional structure (3-D) prediction analysis, it was revealed that TaNBP1 harbors a typical β-propeller feature domain initiated by conserved WD40 repeats (repeats I to VII) (Fig. 2a). Based on distribution of the fusion TaNBP1-GFP detected in transformed tobacco epidermal cells, TaNBP1 was suggested to be located onto positions of cytoplasm membrane and cytosol, given that the GFP signals derived from the fusion were concentrated on these cellular locations (Fig. 2b).

### The expression of *TaNBP1* in response to external N levels

**Fig. 3:**
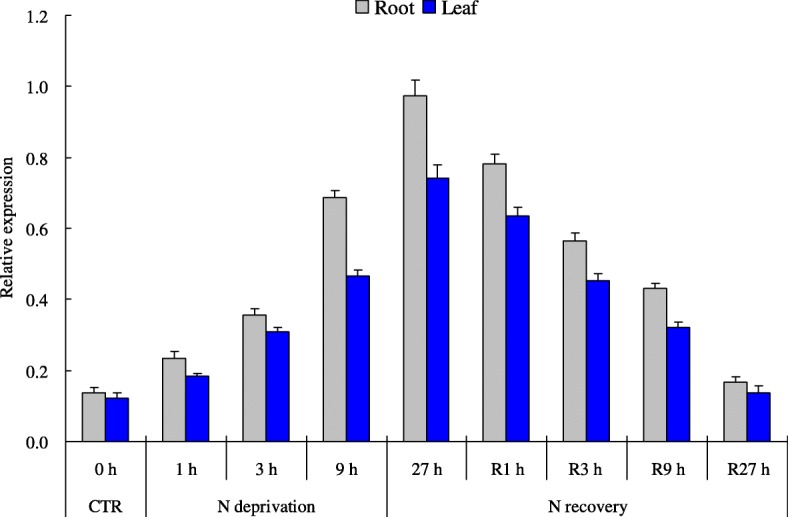
Expression patterns of *TaNBP1* in roots and leaves upon the N starvation stress and N recovery treatment. 0 h, control group. 1 h, 3 h, 9 h, and 27 h, the time points after N deprivation treatment. R1 h, R3 h, R9 h, and R27 h, the time points after N recovery treatment. CTR, control group samples collected before the N-starvation treatment

The expression patterns of *TaNBP1* in tissues of roots and leaves were detected under the varied external N levels. Under N normal condition (16 mM N), the *TaNBP1* transcripts were shown to be low in both roots and leaves. Upon N starvation stress (0.02 mM N), the *TaNBP1* expression in both tissues was gradually upregulated over a 27 h treatment regime, reaching a peak at 27 h after the treatment (Fig. 3). Moreover, the upregulated transcripts of *TaNBP1* in N-deprived tissues were gradually restored upon an N normal recovery treatment, showing that its induced expression was gradually lowered in both tissues along with the N recovery progression (Fig. 3). These results indicate that *TaNBP1* is temporal response to the external N levels.

### *TaNBP1* endows plants improved growth and N acquisition upon N-starvation stress

**Fig. 4:**
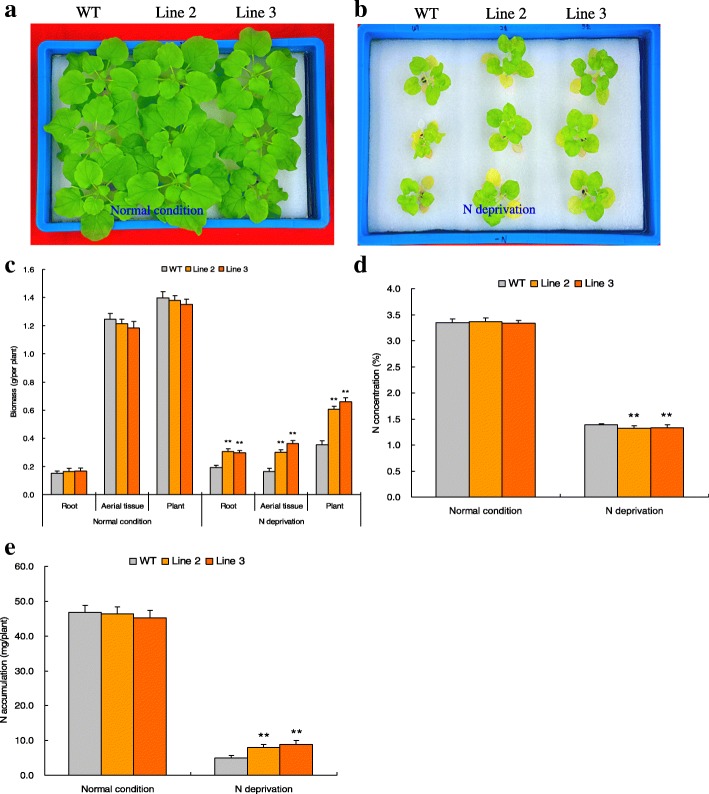
Growth phenotypes, biomass, N concentrations and N amounts in *TaNBP1* overexpression lines under N normal and N starvation treatments. **a** phenotypes after normal N treatment; (**b**) phenotypes after N starvation stress; (**c)** biomass; (**d**) N concentrations; (**e**) N accumulative amounts. WT, wild type. Line 2 and Line 3, two *TaNBP1* overexpression lines. In **(c-e**) error bars indicate SE and symbol ** represents significant difference relative to WT (*P* < 0.01)

Transgene analysis was performed to characterize the *TaNBP1* function in mediating N starvation response. Line 2 and Line 3, two T3 lines possessing more target transcripts (Additional file 1: Figure S3A), were selected and subjected to the N normal and N starvation stress treatments. Under N normal condition, Lines 2 and 3 exhibited comparable phenotypes and biomass with the wild type (WT) (Fig. 4a and c). Under N starvation treatment, however, Lines 2 and 3 both showed enlarged phenotypes, improved root system architecture (RSA), and increased biomass with respect to WT (Fig. 4b and c) (with increase of 54.69 and 59.90% in roots and 83.44 and 122.09% in aerial tissues in Line 2 and Line 3, respectively). These results indicate that *TaNBP1* is crucial in mediating plant N starvation tolerance.

N concentrations in the transgenic lines (Lines 2 and 3) and WT were assessed after the N starvation treatment. No obvious variations in both N concentration and N accumulative amount were observed in transgenic and WT plants under N normal condition (Fig. 4d). In contrast, Lines 2 and 3 exhibited increased accumulative N amounts relative to wild type under N starvation treatment (Fig. 4e, with increase of 63.54 and 79.02% in Line 2 and Line 3, respectively), although the lines displayed comparable N concentrations with WT under the N-deficient condition (Fig. 4d). These results suggest that the *TaNBP1* is involved in enhanced N accumulation that contributes to the plant N starvation tolerance.

### *NtNRT2.2* shows upregulated expression in *TaNBP1* overexpression lines and confers plants enhanced N acquisition upon N starvation stress

**Fig. 5:**
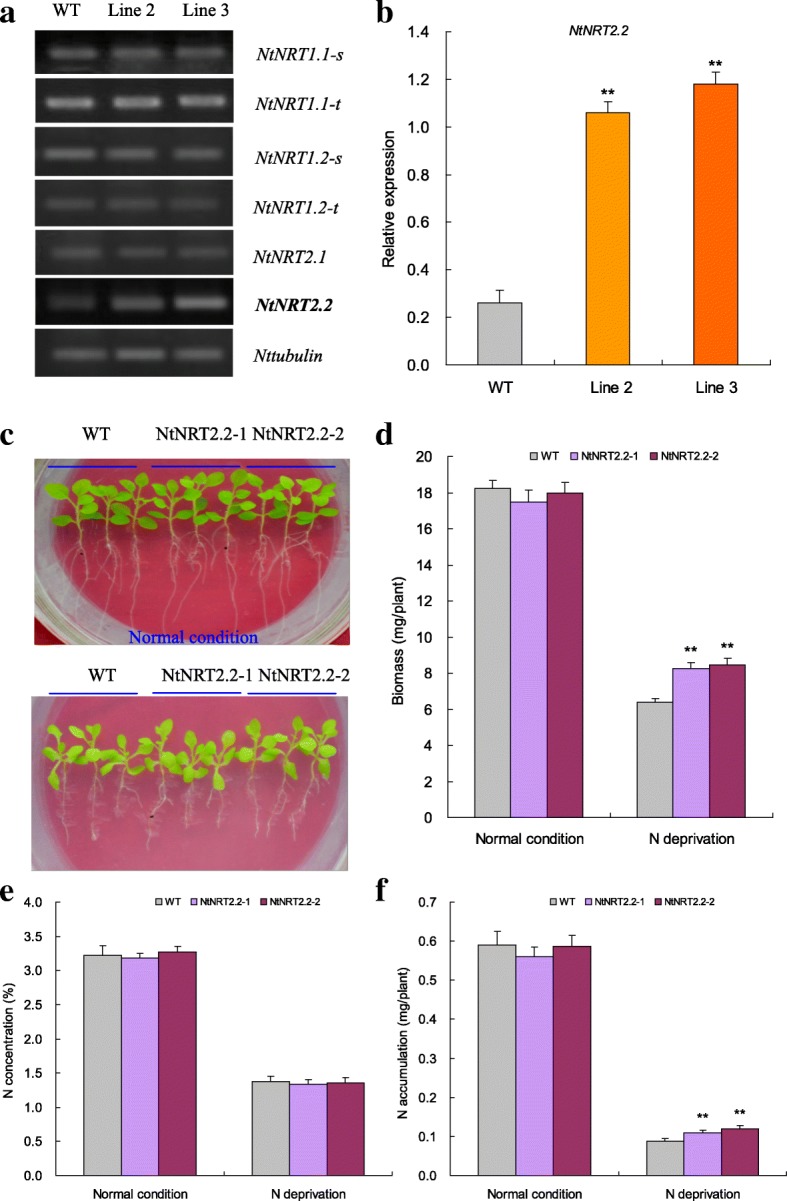
Expression patterns of the NRT genes in N-deprived *TaNBP1* overexpression lines and functional characterization of *NtNRT2.2.* **a** expression patterns of the NRT genes detected by semiquantitative RT-PCR; (**b**) expression patterns of *NtNRT2.2* detected by qRT-PCR; (**c**) seedling phenotypes; (**d**) biomass; (**e**) N concentrations; (**f**) N accumulative amounts. WT, wild type. Line 2 and Line 3, two *TaNBP1* overexpression lines. NtNRT2.2–1 and NtNRT2.2, two *NtNRT2.2* overexpression lines. In (**b**), (**d**), (**e**) and (**f**,) error bars indicate SE and symbol ** represents significant difference relative to WT (*P* < 0.01)

The expression of a suite of tobacco NRT encoding genes, including *NtNRT1.1-s*, *NtNRT1.1-t*, *NtNRT1.2-s*, *NtNRT1.2-t*, *NtNRT2.1*, and *NtNRT2.2*, in the N-deprived transgenic lines (Lines 2 and 3) and WT plants. Among them, the *NtNRT2.2* transcripts were drastically upregulated in lines 2 and 3 with respect to those in WT plants (Fig. 5a, b), contrasting to unaltered expression patterns in other NRT genes examined (i.e., *NtNRT1.1-s*, *NtNRT1.1-t*, *NtNRT1.2-s*, *NtNRT1.2-t*, and *NtNRT2.1*) among the transgenic and WT plants. These results suggest that the transcription of *NtNRT2.2* is under control of *TaNBP1* and the upregulated expression of this NRT gene possibly contributes to increased N accumulation in transgenic lines.

The function of *NtNRT2.2* in improving N accumulation under N starvation stress was evaluated based on transgene analysis. NtNRT2.2–1 and NtNRT2.2–2, two *NtNRT2.2* overexpression lines possessing more target transcripts than others (Additional file 1: Figure S3B), were selected and subjected to N normal and N starvation treatments. Under N normal condition, NtNRT2.2–1 and NtNRT2.2–2 exhibited comparable phenotypes (Fig. 5c), biomass (Fig. 5d), N concentrations (Fig. 5e), and N accumulation (Fig. 5f) with wild type. Under N starvation treatments, however, the transgenic lines displayed improved phenotype (Fig. 5c), increased biomass (Fig. 5d, increase from 29.15 to 32.60%), and elevated N accumulation (Fig. 5f, increase from 25.00 to 36.32%) relative to WT. The increased N amounts in transgenic lines were caused by improved biomass, given that similar N concentrations were observed in transgenic and WT seedlings (Fig. 5e). These results together indicate that *NtNRT2.2* is crucial in mediating plant N acquisition when plants are challenged by N starvation stress.

### *TaNBP1* overexpression improves cellular ROS homeostasis under N starvation conditions

**Fig. 6:**
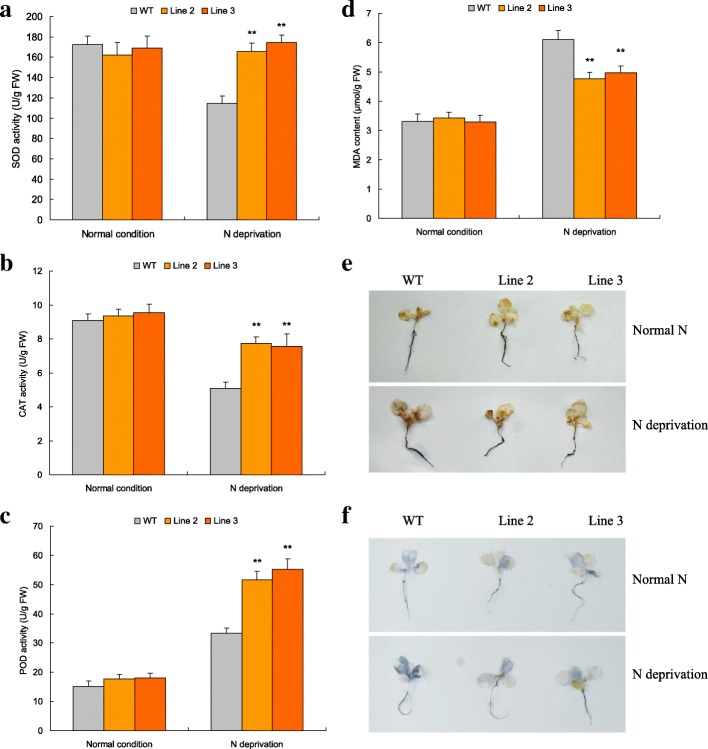
ROS-associated parameters in the N-deprived *TaNBP1* overexpression lines under normal and N starvation treatments. **a** SOD activities; (**b**) CAT activities; (**c**) POD activities; (**d**) MDA contents; (**e**) H_2_O_2_ amounts stained by 3,3 diaminobenzidine (DAB); (**f**) superoxide anion amounts stained by nitroblue tetrazolium (NBT). WT, wild type. Line 2 and Line 3, two *TaNBP1* overexpression lines. In (**a-d**) error bars indicate SE and symbol ** represents significant difference relative to WT (*P* < 0.01)

A set of ROS-associated parameters, including the activities of SOD, CAT, POD, contents of MDA, and the amounts of H_2_O_2_ and superoxide anion in the *TaNBP1* overexpression lines and wild type was assessed after the N normal and N starvation treatments. Under N normal condition, the transgenic lines (Lines 2 and 3) exhibited comparable SOD, CAT, and POD activities and MDA, H_2_O_2_ and superoxide anion contents with WT plants (Fig. 6a to f). Under N starvation treatment, Lines 2 and 3 displayed increased activities of SOD (Fig. 6a, increase from 44.48 to 52.32%), CAT (Fig. 6b, increase from 48.13 to 52.06%), and POD (Fig. 6c, increase from 55.00 to 65.53%), lowered contents of MDA (Fig. 6d, decrease from 18.69 to 27.88%), and reduced H_2_O_2_ and superoxide anion amounts compared with WT (Fig. 6e and f). Therefore, *TaNBP1* confers increased AE activities and improved cellular ROS homeostasis for plants treated by N starvation stress, which contributes to the *TaNBP1*-mediated N starvation tolerance.

### AE gene expression patterns in the N-deprived *TaNBP1* overexpression lines

**Fig. 7:**
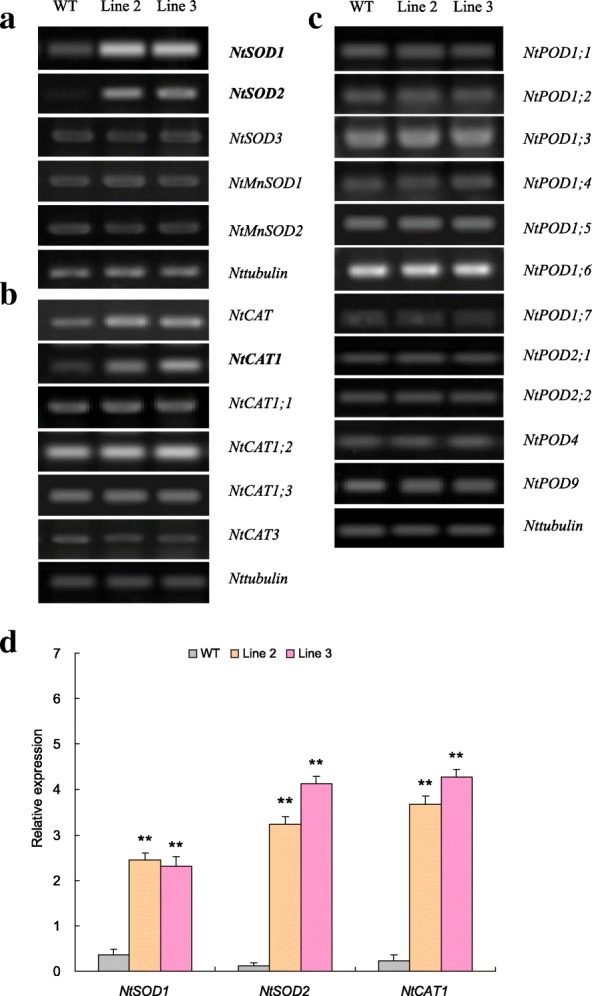
AE gene expression patterns in the N-deprived *TaNBP1* overexpression lines (**a**) expression pattern of SOD genes; (**b**) expression pattern of CAT genes; (**c** )expression pattern of POD genes; (**d**) expression pattern of differential AE genes detected by qRT-PCR. WT, wild type. Line 2 and Line 3, *TaNBP1* overexpression lines. In (**d**) AE gene expression levels in transgenic lines are normalized by the constitutive *Nttubulin* transcripts. Error bars in (**d**) indicate SE and ** represents significant difference relative to WT (*P* < 0.0)

A suite of tobacco AE encoding genes, including five for SOD (i.e., *NtSOD1*, *NtSOD2*, *NtSOD3*, *NtMnSOD1*, and *NtMnSOD2*), six for CAT (i.e., *NtCAT*, *NtCAT1*, *NtCAT1;1*, *NtCAT1;2*, *NtCAT1;3*, and *NtCAT3*), and eleven for POD (i.e., *NtPOD1;1*, *NtPOD1;2*, *NtPOD1;3*, *NtPOD1;4*, *NtPOD1;5*, *NtPOD1;6*, *NtPOD1;7*, *NtPOD2;1*, *NtPOD2;2*, *NtPOD4*, and *NtPOD9*), was subjected to expression evaluation in the N-deprived *TaNBP1* overexpression lines. Among them, two SOD genes (i.e., *NtSOD1* and *NtSOD2*) and one CAT gene (i.e., *NtCAT1*) exhibited significantly upregulated in expression in Line 2 and 3, contrasting to other AE genes examined that showed unaltered transcripts abundance among the transgenic and WT plants (Fig. 7a to d). Therefore, the expression of these differential AE genes is possibly controlled under *TaNBP1* and the upregulated transcription of them possibly impacts on the modified AE activities and cellular ROS homeostasis of the N-deprived *TaNBP1* overexpression lines.

### The differential SOD and POD genes mediate drastically cellular AE activities

**Fig. 8:**
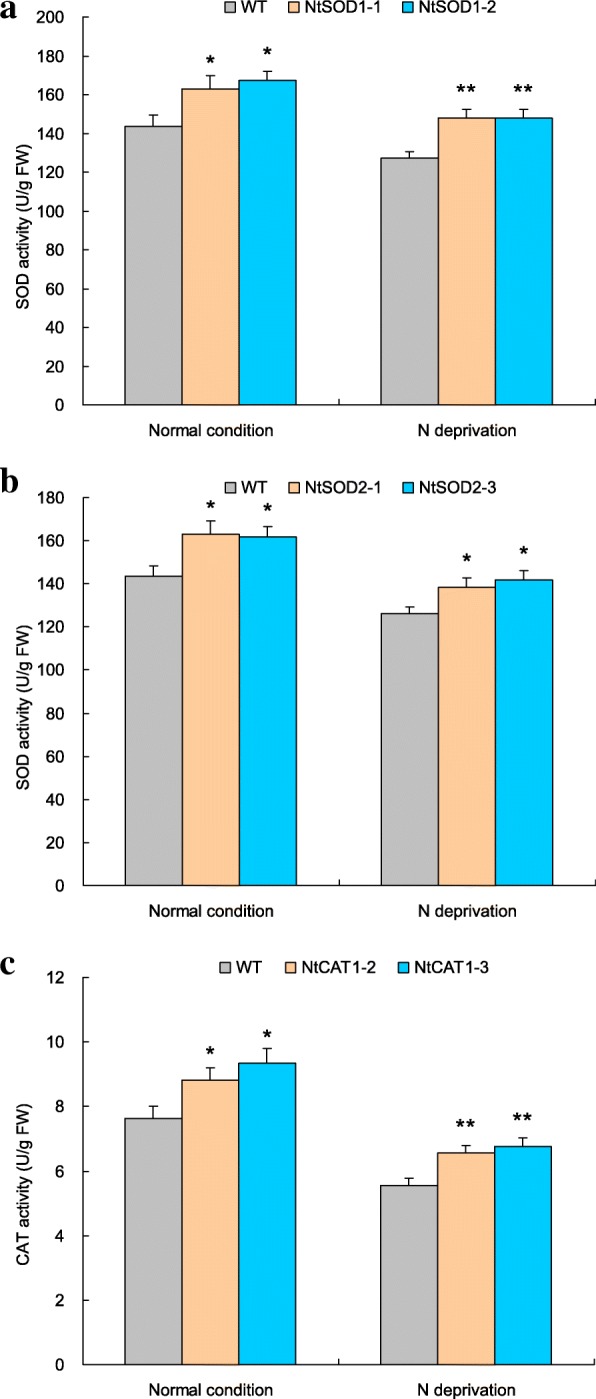
Distinctive AE activities in lines with AE gene overexpression under normal and N starvation treatments. **a** SOD activities in *NtSOD1* overexpression lines; (**b**) SOD activities in *NtSOD*2 overexpression lines; (**c**) CAT activities in *NtCAT1* overexpression lines. WT, wild type. In (**a-c**) error bars indicate SE and symbols* and ** represent significant difference relative to WT at *P* < 0.05 and *P* < 0.01, respectively

The functions of *NtSOD1*, *NtSOD2*, and *NCAT1* in mediating cellular AE activities were characterized based on transgene analysis. Based on target transcripts abundance detection, two lines with strong target gene expression, including SOD1–1 and SOD1–2 for *NtSOD1*, SOD2–1 and SOD2–3 for *NtSOD2*, and CAT1–2 and CAT1–3 for *NtCAT1* (Additional file 1: Figures S4A to S4E), were selected and subjected to the N starvation treatment. Under both N normal and N starvation treatments, all of the transgenic lines showed much higher corresponding AE activities than wild type (Fig. 8a-c), namely, SOD1–1 and SOD1–2 as well as SOD2–1 and SOD2–3 showed enhanced SOD activities (Fig. 8a and b) whereas CAT1–2 and CAT 1–3 displayed increased CAT activities (Fig. 8c) with respect to WT. These results indicate that the differential AE encoding genes involve the regulation of SOD and CAT activities and contribute to the improved cellular ROS homeostasis in the N-deprived *TaNBP* overexpression lines.

## Discussion

The subunits Gα, Gβ and Gγ involved in assembly of G-proteins are conserved in all eukaryotes. The members in family constituting these subunits are generally limited in plant species. For example, only a subunit Gα encoding gene (*GPA1*) [25], a subunit Gβ encoding gene (*AGB1*) and a set of genes encoding subunit Gβ-like [26], and three subunit Gγs genes (*AGG1*, *AGG2* and *AGG3*) that encode Gγ1, Gγ2 and Gγ3, respectively [27], have been identified in Arabidopsis. Characterization on the G-protein subunits has validated their critical roles in the modulation of diverse biological processes associated with plant growth, development and stress responses [9, 10].

Subunit Gβ of G-proteins is involved in regulation of various physiological processes in plants, including to mediate plant pathogen *P. syringae* defensiveness [28], modulate plant flowering initiation [14], regulate seed germination, ABA-mediated stomatal opening and inward K^+^-channel activation [29], and modulate plant phenotype and silique development [30, 31]. In this study, characterization on the wheat Gβ subunit TaNBP1 indicated that it bears conserved WD40 repeats, which involve the 3-D structure establishment of the NBP1 family proteins. Our analysis on detecting the GFP distribution at subcelluar level from TaNBP1-GFP fusion revealed that the signals were concentrated onto cytoplasm membrane and cytosol, suggesting that TaNBP1 targets to these positions after sorted from endoplasmic reticulum, where it established the heterotrimic G-protein with subunits Gα and Gγ.

Distinct N signaling is elicited upon N starvation stress, which initiates transcriptional alteration on a large set of the regulatory and the functional genes [32]. A suite of genes involving plant N starvation stress response, including those coding for NRT proteins [32], ammonia transporters [33], Dof1 transcription factor [5], glutamine synthetase [34], and auxin receptor (AFB3) [35], shows modified transcription patterns upon the varied external N levels. In this study, we found that the expression patterns of *TaNBP1* exhibited variation under modified N input conditions, showing that the *TaNBP1* transcripts were gradually increased in roots and leaves over a 27 h regime under N starvation treatment. Moreover, the upregulated expression of this wheat gene was gradually downregulated once the N-deprived plants were resubjected to a N normal recovery treatment. These expression patterns suggest that *TaNBP1* is involved in plant N starvation response. Previously, a *cis*-acting regulatory element referred to as NRE was shown to be critical in transcriptional regulation of a suite of N-responsive genes, including those enconding nitrite reductase (NIR) and NRT proteins in Arabidopsis, such as *NRT2.1* and *NRT2.2* [32]. Further characterization of the *cis*-regulatory elements underlying N starvation response of *TaNBP1* can help understand its transcriptional mechanism upon low-N stress.

Functional characterization on subunit Gβ has revealed its various biological roles, including modulation of hypocotyl cell division [30], seed germination [31], seedling establishment and root elongation [31, 36], stomatal opening and inward K^+^-channel responses in the guard cells [18]. In this study, the observation on N deprivation response prompted us to characterize the function of *TaNBP1* in regulating plant N starvation tolerance. Under N starvation treatment, the lines with *TaNBP1* overexpression exhibited improved phenotypes, increased N accumulative amounts, and higher plant biomass with respect to the wild type (Fig. 4a-e). The significant improvement on N uptake and plant dry matter production under low-N stress in transgenic lines suggests that *TaNBP1* is essential in modulating the plant adaptation to N starvation stress. It is also valuable in crop genetically engineering with high NUE under N-deficient conditions.

Low-affinity transport system (LATS) and high-affinity transport system (HATS) are the two major N uptake systems in plant species and other eukaryotes, which play crucial roles in the mediation of plant N acquisition and internal N translocation across tissues upon various N input conditions [37]. Of which, HATS is constisted of the high-affinity nitrate transporters encoded by NRT2 family genes, which are functional in plant N uptake under N starvation stress [38]. Previously, based on mutants analysis, the functions of a set of NRT2 family members in mediating N acquisition under low-N stress were characterized. For example, mutants with knockout of *NRT2.1*, *NRT2.2*, *NRT2.4*, and *NRT2.7*, the Arabidopsis NRT2 family genes, show drastic reduction on root nitrate absorption capacity [39–41]. In this study, we analyzed the expression patterns of a suite of NRT encoding genes to address weather they were involved in the *TaNBP1*-mediated improvement on N uptake under N starvation stress. Our results indicated in contrast to other NRT genes examined that showed unaltered transcription in the N-deprived *TaNBP1* overexpression lines and wild type, one NRT family member referred to as *NtNRT2.2* showed upregulated in expression in transgenic lines relative to that of wild type. Transgene analysis on *NtNRT2.2* indicated that the lines with overexpression of this NRT gene conferred plants improved phenotype and increased N accumulative amounts (Fig. 5c, d, f). These findings suggested that *NtNRT2.2* contributes to plant N uptake under the low-N stress. Therefore, the *TaNBP1*–mediated N starvation tolerance is associated with the upregulated expression of *NtNRT2.2*, whose transcription under *TaNBP1* control impacts on the plant N accumulation, phenotype behavior, and biomass production once plants are challenged by the N starvation stress.

Root system architecture (RSA) impacts largely on the water acquisition and the inorganic nutrient uptake from growth media. In addition to the NRT protein mediated N uptake, the RSA modulated by low-N stress also drastically impacts on the plant N uptake, whose establishment has been reported to be associated with the involvement of distinct NRT genes. For example, the Arabidopsis *NRT2.1* involves the physiological processes of initiation and extension of the primary and secondary roots, aside from its role in regulating NO_3_^−^ uptake [32]. In this study, our analysis on RSA characterization in lines with overexpression of *TaNBP1* or *NtNRT2.2* revealed that they all displayed enlarged RSA system under N starvation treatment compared with wild type (Figs. 4c and 5c). This finding suggested the involvement of *TaNBP1* in mediating RSA establishment, which was possibly accomplished through its transcriptional regulation on *NtNRT2.2*. Previously, the RSA behaviors modulated upon altered N levels was shown to be associated with auxin, a type of the phytohormones. The corresponding evidence was provided in maize (*Z. mays*), whose root growth is inhibited under high nitrate conditions and the inhibition intensity is correlated with the reduction on auxin concentration in root tips. Alleviation of transduction of the auxin signaling from shoot to root then involves modulation of the RSA behavior [42]. Moreover, several investigations have indicated the internal connections between the G-proteins, NRT proteins, and the cellular auxin transportation characterization. For example, the Arabidopsis Gβ (also designated as AGB1) mediates acropetal transportation of auxin within the central cylinder, affecting the action of auxin in epidermis and/or cortex and further modulating RSA establishment [43]; Arabidopsis NRT1.1 regulates negatively cellular auxin transportation and modulates positively the auxin-mediated RSA establishment under N starvation conditions [38]. These findings together suggest the synergic action mechanism underlying G-protein, NRT protein, and auxin in regulating the RSA behavior. Further characterization on RSA behaviors regulated by *TaNBP1* and *NtNRT2.2* can help understanding of the root phenotype formation of plants.

Cellular ROS overproduction upon diverse stresses results in damage of proteins, lipids, and nucleic acid, leading ultimately to cellular injury or cell death [44]. On the other hand, plants have evolved a suite of corresponding protection strategies (i.e., enzymatic and non-enzymatic antioxidative systems) to protect themselves from the oxidative damage [45]. Antioxidant enzymes (AE) such as SOD, CAT, and POD are critical mediators in detoxification of ROS initiated by environmental stressors. Recently, it has been validated that the transcription of distinct AE genes is regulated under control of the G-protein subunits. For example, the transcripts of the CAT gene in rice (*O. sativa*) are regulated by RGG1, a γ subunit of G-protein; overexpression of *RGG1* confers plants enhanced CAT activities and improved salt tolerance [46]. This finding suggests the putative connection between the subunits of G-protein and cellular ROS homeostasis of plants. In this study, based on assessment of the ROS-associated parameters in N-deprived *TaNBP1* overexpression lines, we revealed the function of *TaNBP1* in mediating cellular ROS homeostasis under N deprivation. Our results indicated that the *TaNBP1* overexpression lines exhibited increased SOD, CAT, and POD activities, lowered MDA contents, and reduced H_2_O_2_ and superoxide anion accumulative amounts (Fig. 6a to f) with respect to those of wild type. Further transgene analysis on a set of differental AE genes, including *NtSOD1*, *NtSOD2*, and *NtCAT1*, three AE genes showing significantly upregulated in expression in lines with *TaNBP1* overexpression, confirmed the roles of them in modulating AE activities. These results suggest that the TaNBP1-mediated N starvation tolerance was also associated with the gene function in detoxification of cellular ROS. Transcriptional mechanisms of the differential AE genes underlying *TaNBP1* regulation are needed to be further characterized.

## Conclusion

Our investigation indicates that *TaNBP1* is transcriptional response to external N levels. Overexpression of *TaNBP1* confers plants improved phenotype, N accumulative amount, and biomass production under N starvation conditions, indicating that *TaNBP1* acts as one of essential regulators in plant adaptation to the N-starvation stress. *TaNBP1*–mediated plant N-starvation tolerance is closely associated with the gene functions in improving N acquisition, RSA establishment, and cellular ROS homeostasis through transcriptional regulation of NRT gene *NtNRT2.2* and distinctive AE genes such as *NtSOD1*, *NtSOD2*, and *NtCAT1*. Overexpression of these differential genes significantly regulates plant N uptake, root architecture establishment, and AE activities. *TaNBP1* can be used as one of valuable target genes in crop genetically engineering with high NUE under the N-saving cultivation conditions.

## Additional file

Additional file 1: **Table S1.** PCR primers used in this study. **Figure S1.** The full length cDNA of *TaNBP1* and the corresponding translated amino acids. The start codon ATG and the termination codon TAG of *TaNBP1* are labeled by red background. Seven conserved WD40 repeat domains (I to VII) consisting of a sevenfold β-propeller in TaNBP1 are highlighted by blue background. **Figure S2.** Phylogenetic relations between *TaNBP1* and its homologous genes from various plant species. **Figure S3.** Target gene transcripts in lines overexpressing *TaNBP1* and *NtNRT2.2* **a**, *TaNBP1* transcripts in transgenic lines; **b**, *NtNRT2.2* transcripts in transgenic lines. WT, wild type. Line 1 to Line 7, independent transgenic lines with *TaNBP1* overexpression. NtNRT2.2–1 to NtNRT2.2–6, independent lines with *NtNRT2.2* overexpression. In **a**, *TaNBP1* **e**xpression levels in transgenic lines are normalized by the constitutive *Tatubulin* transcripts. In **b**, *NtNRT2.2* expression levels in transgenic lines are normalized by the constitutive *Nttubulin* transcripts. Internal standard reference genes are set an expression level of 1. **Figure S4.** Target gene transcripts in lines overexpressing differential AE genes **a**, *NtSOD1* transcripts in transgenic lines; **b**, *NtSOD2* transcripts in transgenic lines; **c**, *NtCAT1* transcripts in transgenic lines; WT, wild type. Expression levels of the AE genes in transgenic lines are normalized by the constitutive *Nttubulin* transcripts whose expression level is set as 1. (DOC 162 kb)

## Abbreviations

AE: Antioxidant enzyme
CAT: Catalase
MS: Murashige and skoog
NRT: Nitrat transporter
ORF: Open reading frame
POD: Peroxidase
ROS: Reactive oxygen species
WT: Wild type
