# Supplementary material for: TaNBP1, a guanine nucleotide-binding subunit gene of wheat, is essential in the regulation of N starvation adaptation via modulating N acquisition and ROS homeostasis
Source: BMC Plant Biol. 2018 Aug 13;18:167. doi: 10.1186/s12870-018-1374-6 (PMC6090633; doi:10.1186/s12870-018-1374-6)
Supplement: Supplementary file 1 — Table S1. PCR primers used in this study. Figure S1. The full length cDNA of TaNBP1 and the corresponding translated amino acids. The start codon ATG and the termination codon TAG of TaNBP1 are labeled by red background. Seven conserved WD40 repeat domains (I to VII) consisting of a sevenfold β-propeller in TaNBP1 are highlighted by blue background. Figure S2. Phylogenetic relations between TaNBP1 and its homologous genes from various plant species. Figure S3. Target gene transcripts in lines overexpressing TaNBP1 and NtNRT2.2 a, TaNBP1 transcripts in transgenic lines; b, NtNRT2.2 transcripts in transgenic lines. WT, wild type. Line 1 to Line 7, independent transgenic lines with TaNBP1 overexpression. NtNRT2.2–1 to NtNRT2.2–6, independent lines with NtNRT2.2 overexpression. In a, TaNBP1 expression levels in transgenic lines are normalized by the constitutive Tatubulin transcripts. In b, NtNRT2.2 expression levels in transgenic lines are normalized by the constitutive Nttubulin transcripts. Internal standard reference genes are set an expression level of 1. Figure S4. Target gene transcripts in lines overexpressing differential AE genes a, NtSOD1 transcripts in transgenic lines; b, NtSOD2 transcripts in transgenic lines; c, NtCAT1 transcripts in transgenic lines; WT, wild type. Expression levels of the AE genes in transgenic lines are normalized by the constitutive Nttubulin transcripts whose expression level is set as 1. (DOC 162 kb) [file 12870_2018_1374_MOESM1_ESM.doc]

**Table S1** PCR primers used in this study

| **Purpose** | **Accession number** | **Forward primer (5´-)** | **Reverse primer (5´-)** |
| --- | --- | --- | --- |
| *TaNBP1* expression | AK332651 | AGCTCCGCCGCCCCAATCT | AACCTCTCTGGCCAGCAAGAC |
| *Tatubulin* expression | U76558 | AGAACACTGTTGTAAGGCTCAAC | GAGCTTTACTGCCTCGAACATGG |
| *TaNBP1* localization cassette | AK332651 | TTTGTCGACAATGGCGAACATGCAGGAG | TTTCCATGAACGCGTATCCGGCGAACC |
| *TaNBP1* overexpression cassette | AK332651 | TTTCCATGGCGAACATGCAGGAG | TTTGGTGACCCCTACGCGTATCCGG |
| *Nttubulin* expression | U91563 | TACACAGGGGAAGGAATGG | CTCGAAACCAACGGTATC |
| *NtNRT1.1-s* expression | AB102805 | TACCGGTTTGTCGACGTGTC | TCTCTTCTCCTTGTACACATAC |
| *NtNRT1.1-t* expression | AB102806 | CCGGCTTCATTGACACTCTT | CCTCTTCTCCTTGTACACATAC |
| *NtNRT1.2-s* expression | AB102807 | GGGTTATCGTTCCCATTTGTCGT | TCAGCAAGTCTCTTCTCCTTGT |
| *NtNRT1.2-t* expression | AB102808 | GCCCTAACAGAGGTTAAGAGG | TCCCCATTTCAGCAAGTCTC |
| *NtNRT2.1* expression | AJ557583 | TAGCCGTCACATTCATGATCCTT | GATCGGCAGTTCTCGGCGAA |
| *NtNRT2.2* expression | AJ557584 | CGTCGATCGTTAGGTATAATC | ATTAACTACTCACACTTGGGTAA |
| *NtNRT2.2* overexpression cassette | AJ557584 | AAACCATGGTTGATATTGAAGGA | AAACACGTGAGACTCAAGCTCAGA |
| *NtSOD1* expression | KJ874395 | GTGGACATGTCGTGTCAAGG | TTCTCACCAACTCCTGCACTT |
| *NtSOD2* expression | KF724056 | CATCACAGAGCTTATGTCGACA | CTAGAACTGACTGCTTCCCA |
| *NtSOD3* expression | EU123521 | ATGTCACGGGACCACATTAC | AACCCTTCCACCAGCATTTC |
| *NtMnSOD1* expression | X14482 | TTGGGCTATCGACACTAACTTT | TCAGCCAGCGACTACATGCA |
| *NtMnSOD2* expression | AB093097 | GACGGACCTTAGCAACAGGG | ACCAATGGGTCCTGATTAGCAG |
| *NtCAT* expression | EF532799 | CAAGGATCTCTACGACTCGATT | CTTGAGGGCAAATAATCCACCT |
| *NtCAT1* expression | NTU07627 | GTCTCAGGCTGACAAGTCTT | ACGGAAGACAGAGTAGCAGC |
| *NtCAT1;1* expression | NTU93244 | TCCTGCTAATGCTCCAAAGTGT | AATGCATATGTATTAGGAATGCTC |
| *NtCAT1;2* expression | HF564632 | GGTATCGACTTGGACCAAACTA | GGTCTCACATTAAGCCTAGAAG |
| *NtCAT1;3* expression | HF564631 | TTGCAGCCGGTGGGAAGATT | GGTCTCACATTAAGCCTAGAAG |
| *NtCAT3* expression | HF564633 | GTCTTGGGCCAAACTATCTGCA | TCAGCTTCACATTGTGGGCC |
| *NtPOD1;1* expression | L02124 | GGAATTTGTCCTCAAGGTGGAA | CTTATTGGAATTGCCATTTCAGC |
| *NtPOD1;2* expression | AB044154 | CTGACATGGTCTGTGCCTAC | TCAGTTGATAGCAGAGCAAACTT |
| *NtPOD1;3* expression | AB044153 | AAGATCTTGTCGCTCTTACTGG | AATTGGATTTTCCAGCTTGCG |
| *NtPOD1;4* expression | D11396 | TGCTGGTAGTCAAAGTCAGTTTT | CCCATGTTGAACACGTTCTTACC |
| *NtPOD1;5* expression | AB178953 | AACAGCAACAACGTTAACCCAGC | TTAATTTTGGACCACATTCAGGA |
| *NtPOD1;6* expression | AB027753 | TCAACTCCACTGGTGGCCCT | AATTCGATTTTGCAGCTTGCGC |
| *NtPOD1;7* expression | AB027752 | GCCCAAGAAGTTCAGGCTCA | ATACAAATACAGTCCTTTACTCG |
| *NtPOD2;1* expression | AB178954 | AGGGGAAAAGACCTCACCAC | AGTTTCCCATCTTGATCATAGCA |
| *NtPOD2;2* expression | KF701483 | AGACAGTGAGTATGCAGCTAA | AAAAAAGCTGCCCTTGGCACC |
| *NtPOD4* expression | AY032675 | AGACTCAAAGATAGCAAACCTCA | CTTCCTGATGTCACCCTTGA |
| *NtPOD9* expression | AY032674 | CACCACCTTCATTCAACGCTA | ACATCTCAGACAAAACACTTGTC |
| *NtSOD1*  overexpression cassette | KJ874395 | AAACCATGGCATTTTTGAGGTCA | AAAGGTAACCTCAAAGTTTGTTGGTTGC |
| *NtSOD2*  overexpression cassette | KF724056 | TTTCCATGGTGATGGCCGCTACT | TTTGGTAACCTCAAGCTGTTGCTGCTTT |
| *NtCAT1* overexpression cassette | NTU07627 | TTTCCATGGATCTCTCTAAGTTT | TTTGGTAACCCAACTTGAGACGTTTTCC |
| *NtPOD1;5* overexpression cassette | AB178953 | TTTCCATGGCTTTTCGTTTGAGT | TTTGGTAACCAACCAGTGATTCTTTTAT |
| *NtPOD1;6* overexpression cassette | AB027753 | TTTCCATGGAGTACTATCACCATT | TTTGGTAACCATAAAAGGGTACAATATC |


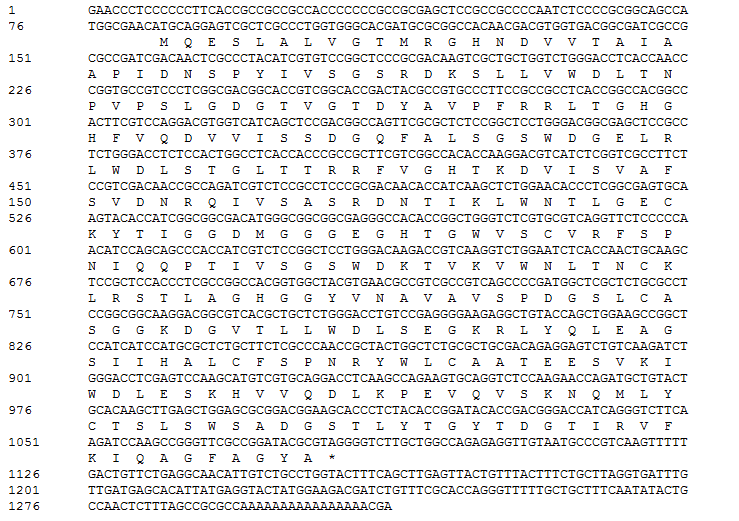


**I**

**II**

**III**

**IV**

**V**

**VI**

**VII**

**Figure S1.** The full length cDNA of *TaNBP1* and the corresponding translated amino acids

The start codon ATG and the termination codon TAG of *TaNBP1* are labeled by red background. The seven conserved WD40 repeat domains (I to VII) consisting of a sevenfold β-propeller are highlighted by blue background.


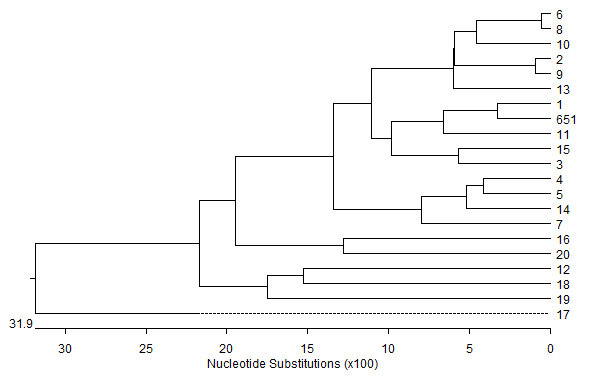


*Z. mays G-protein β like*（EU962678.1）

*Z. mays G-protein β like*（NM_001143328）

*S.bicolor* protein (XM_002440157)

*Z.mays* cDNA (BT055706)

*Z.mays* cDNA (NM_001153504)

*S.italica G-protein β likeA* (XM_004961250)

*H.vulgare* protein (AK359815)

*TaNBP1* (AK332651)

*B.distachyon G-protein β likeA* (XM_003567896)

*O.brachyantha* *G-protein β likeB* (XM_006654687)

*O.sativa* cDNA (CT833917)

*O.sativa* RACK (D38231.1)

*O.sativa G-protein β* (EF576355.1)

*O.brachyantha* *G-protein β like A* (XM_006644498.1)

*P.edulis* cDNA (FP093851.1)

*P. dactylifera* *G-protein β l*ike A (XM_008814393.1)

*E.guineensis G-protein β like A* (XM_010918027)

*F.vesca* *G-protein β* (XM_004299500.2)

*E.grandis G-protein β* (XM_010038637)

*T.hassleriana* *G-protein β likeB* (XM_010550775.1)

*M.acuminata* *G-protein β like* A (XM_009382228.1)

Identity

78.1%

75.8%

77.3%

79.5%

60.2%

79.4%

93.8%

100%

87.2%

78.1%

83.6%

77.8%

72.1%

78.0%

77.4%

63.1%

75.8%

59.2%

54.4%

57.0%

60.2%

**Figure S2.** Phylogenetic relations between *TaNBP1* and its homologous genes from various plant species

**a**

**b**

**Figure S3.** The target gene transcripts in lines overexpressing *TaNBP1* and *NtNRT2.2*

**a**, the *TaNBP1* transcripts in transgenic lines; **b**, the *NtNRT2.2* transcripts in transgenic lines. WT, wild type. Line 1 to Line 7, independent transgenic lines with *TaNBP1* overexpression. NtNRT2.2-1 to NtNRT2.2-6, independent lines with *NtNRT2.2* overexpression. In **a**, the *TaNBP1* **e**xpression levels in transgenic lines are normalized by the constitutive *Tatubulin* transcripts. In **b**, the *NtNRT2.2* expression levels in transgenic lines are normalized by the constitutive *Nttubulin* transcripts. Internal standard reference genes are set an expression level of 1.

**a**

**b**

**c**

**Figure S4.** Target gene transcripts in lines overexpressing differential AE genes

**a**, the *NtSOD1* transcripts in transgenic lines; **b**, *NtSOD2* transcripts in transgenic lines; **c**, the *NtCAT1* transcripts in transgenic lines; WT, wild type. Expression levels of the AE genes in transgenic lines are normalized by the constitutive *Nttubulin* transcripts whose expression level is set as 1.
